# Cognitive Outcomes for Essential Tremor Patients Selected for Thalamic Deep Brain Stimulation Surgery Through Interdisciplinary Evaluations

**DOI:** 10.3389/fnhum.2020.578348

**Published:** 2020-12-11

**Authors:** Jacob D. Jones, Tatiana Orozco, Dawn Bowers, Wei Hu, Zakia Jabarkheel, Shannon Chiu, Adolfo Ramirez-Zamora, Kelly Foote, Michael S. Okun, Aparna Wagle Shukla

**Affiliations:** ^1^Department of Psychology, California State University, San Bernardino, CA, United States; ^2^Department of Neurology, Fixel Institute for Neurological Diseases, University of Florida, Gainesville, FL, United States; ^3^Department of Neurosurgery, University of Florida, Gainesville, FL, United States

**Keywords:** neuropsychology, DBS (deep brain stimulation), essential tremor, working memory, cognitive

## Abstract

**Objective**: Deep brain stimulation (DBS) targeted to the ventral intermediate (VIM) nucleus of the thalamus is effective for motor symptoms in essential tremor (ET), but there is limited data on cognitive outcomes. We examined cognitive outcomes in a large cohort of ET DBS patients (pre-DBS and 1+ year after DBS).

**Methods**: In a retrospective analysis, we used repeated-measures ANOVA testing to examine whether the age of tremor onset, age at DBS surgery, hemisphere side implanted with lead, unilateral vs. bilateral implantations, and presence of surgical complications influenced the cognitive outcomes. Neuropsychological outcomes of interest were verbal memory, executive functioning, working memory, language functioning, visuospatial functioning, and general cognitive function.

**Results**: We identified 50 ET DBS patients; 29 (58%) males; the mean age of tremor onset was 35.84 (±21.50) years with a median age of 38 years. The mean age at DBS was 68.18 (±10.07) years. There were 37 unilateral 30 left, seven right, and 13 bilateral brain implantations. In the subgroup analysis, there was a significant interaction between assessment (pre vs. post) and age of tremor onset (<38 vs. >38 years); *F*_(1,30)_ = 4.47; *p* = 0.043 for working memory. The *post hoc* testing found improvements for younger onset ET. Similarly, there was a significant interaction between assessment (pre vs. post) and complications vs. no complications subgroups; *F*_(1,45)_ = 4.34; *p* = 0.043 for verbal memory with worsening scores seen for ET patients with complications. The remaining tests were not significant.

**Conclusion**: In this large cohort of ET patients with (>30% improvements), DBS was not accompanied by a significant decline in many cognitive domains. These outcomes were possibly related to the selection of patients with normal cognitive functioning before surgery, unilateral DBS implantations for the majority, and selection of patients with optimal response to DBS.

## Introduction

Deep brain stimulation (DBS) directed to the ventral intermediate (VIM) nucleus region of the thalamus is an efficacious intervention for the motor symptoms in essential tremor (ET; Wilkes et al., 2020). ET is associated with cognitive impairments evident on tests of attention, verbal fluency, and response inhibition (Bermejo-Pareja and Puertas-Martin, 2012). DBS has been increasingly applied to treat many movement disorders, including Parkinson’s disease, dystonia, and Tourette’s syndrome. DBS settings used for the treatment of motor symptoms can be accompanied by a further decline in cognition, and a small decrement in cognitive function after DBS can have demonstrable effects on the quality of life (Tröster and Massano, 2015). While DBS in Parkinson’s disease has shown a consistent decline in verbal fluency (Okun et al., 2009; Demeter et al., 2017; Mehanna et al., 2017), the data for ET is sparse and in some cases conflicting. In one study, VIM DBS impaired the word output dynamics during verbal fluency tasks (Ehlen et al., 2017), whereas Fields et al. (2003) observed that there was a significant improvement in general cognitive assessment, verbal memory and visuoperceptual functions following DBS. However, these studies did not evaluate the baseline factors that influenced cognitive performance following surgery. We retrospectively examined the neuropsychology outcomes in a relatively large cohort of ET patients presenting to our center for VIM DBS surgery. We sought to examine whether factors including the age of onset for ET, the hemisphere side implanted with the DBS lead, unilateral vs. bilateral implantation procedures, and the presence of surgical complications influenced cognitive performance following DBS surgery. We further examined whether disease duration, age at DBS surgery, tremor severity, and the electrical energy used for DBS programming predicted cognitive outcomes.

## Materials and Methods

The Institutional Review Board of the University of Florida that follows the ethical standards according to the Declaration of Helsinki approved our plans for retrospective analysis of data. We extracted outcomes for neuropsychology assessment performed at about 1 year after DBS electrode implantation to compare against the baseline. Participants in this study followed standard procedures followed at the University of Florida for DBS surgery. First, medication-refractory ET patients were evaluated by an interdisciplinary team of Neurology, Neurosurgery, Psychiatry, Neuropsychology, Physical, Occupational, and Speech therapy. Patients were deemed eligible for DBS if the tremor impacted the activities of daily living, and significant surgical or psychiatric comorbidity (moderate to severe depression), balance disorder, speech dysfunction, and dementia were not present. When patients had substantial bilateral tremors impacting the manual dexterity, staged bilateral DBS procedure was offered if safe from a cognitive and gait/balance perspective. These staged procedures included unilateral implantation followed 6 months later by the second sided implant. If patients opted for only one DBS lead, we targeted either their dominant hand or worse hand tremor.

For patients who underwent staged bilateral DBS, we extracted cognitive data that was recorded 1 year after the second electrode was implanted. DBS electrodes (model 3387, Medtronic, Minneapolis, MN, USA) were implanted upon identification of the target with imaging techniques involving CT and stereotactic magnetic resonance imaging (MRI) using a fusion process. Target identification was further refined with intraoperative microelectrode recording. Once the lead was implanted, intraoperative stimulation was performed to ensure there was adequate tremor control at lower voltages and side effects such as muscle contraction and speech deficits arising from internal capsule stimulation were only present at higher voltages. The fusion of postoperative helical CT scans with preoperative MRI scans was used to localize the entry zones of the DBS electrode in the cortex as well as the active contact in the brain. Following the lead implantation procedure, about 30 days later under general anesthesia, a pulse generator connected to an electrode lead was placed subcutaneously in the subclavicular region. DBS programming was initiated about 2–4 weeks after the pulse generator surgery. Lead contact and optimal stimulation parameters were determined based on empirical monthly programming sessions performed on an outpatient basis.

The following methods that are specifically relevant to our study were applied. We included ET DBS subjects who underwent neuropsychology assessments before DBS (pre-DBS) and 1–2 years after surgery (post-DBS). These assessments were performed while the patients continued tremor medications, and DBS was kept “on” for the post-surgery neuropsychology assessments. The exclusion criteria were: neurological disorder besides ET suboptimally placed DBS lead, defined by <30% improvement in tremor at 6 months after implantation of the lead, neuropsychology assessment before the surgery was performed at other institutions, and patients who reported a history of prior thalamotomy.

All participants consented to have their data stored in an IRB approved database at the Norman Fixel Institute for Neurological Diseases. In addition to neuropsychology measures, we extracted demographics, handedness, education status, disease-related measures, side of surgery for DBS implantation, and the stimulation parameters used for DBS programming in this cohort. Tremor severity was assessed with the Fahn-Tolosa-Marin Tremor Rating Scales (TRS; Fahn and Marin, 1993). Beck Depression Inventory-II and State-Trait Anxiety Inventory scale scores were also collected as measures of depression and long-standing anxiety (Beck and Brown, 1996; Spielberge, 2010). We also recorded the total electrical energy delivered (TEED) to the optimal lead contact as calculated with the standard formula at the time of follow-up neuropsychology assessment (post-DBS; Fakhar et al., 2013).

Cognitive measures consistent with our past experience involving neuropsychological assessments were grouped into six cognitive domains: verbal memory, executive functioning, working memory, language, visuospatial functioning, and global cognitive function (Sheline et al., 2006). The verbal memory domain consisted of the delayed free recall scores of the Logical Memory Stories from the Wechsler Memory Scale-III and the delayed free recall of the Hopkins Verbal Learning Test-Revised. Tests of executive functioning included the Trails Making Test, part B, the color-word trial of the Stroop Color-Word Test (Golden version), and a letter fluency task (Controlled Oral Word Association Test; COWA). Working memory tests included the Forward Span and Backward Span trials of the Digit Span subtest from the Wechsler Adult Intelligence Scale-III. Language tests consisted of the Boston Naming Test (total items correctly named) and a semantic fluency test. The Judgment of Line Test and the Benton Facial Recognition Test were used as tests of visuospatial functioning. Finally, general cognitive functioning was assessed with the Dementia Rating Scale-II (DRS-II). Raw scores were normed on age and gender-based on test-specific manuals or previously published norms and converted to a Z-metric (Heaton, 2004). The individual test *z*-scores were averaged to form the domain composites (Jiménez-López et al., 2017).

### Statistical Analyses

We used IBM SPSS Statistics version 26 to analyze the data. We examined whether cognitive outcomes after surgery in each domain of assessment differed according to the baseline characteristics including the age of tremor onset, age at DBS surgery, disease duration, baseline TRS score, unilateral vs. bilateral DBS, right DBS vs. left DBS (amongst the unilateral DBS patients); the amount of TEED and complications during and immediately after surgery. We used univariate regression to determine the effects of age at DBS surgery, disease duration, baseline tremor severity, and TEED and we employed repeated measures ANOVA to determine the main and interaction effects of assessment time (pre- vs. post-DBS) and assignment to subgroup (younger vs. older onset ET; unilateral vs. bilateral; right hemisphere vs. left hemisphere; complications vs. no complications). We set the significance to *p* < 0.05, and significant effects were further probed using Bonferroni-corrected pairwise comparisons. We employed a *t*-test for comparing motor and mood changes with DBS.

## Results

**Figure 1:**
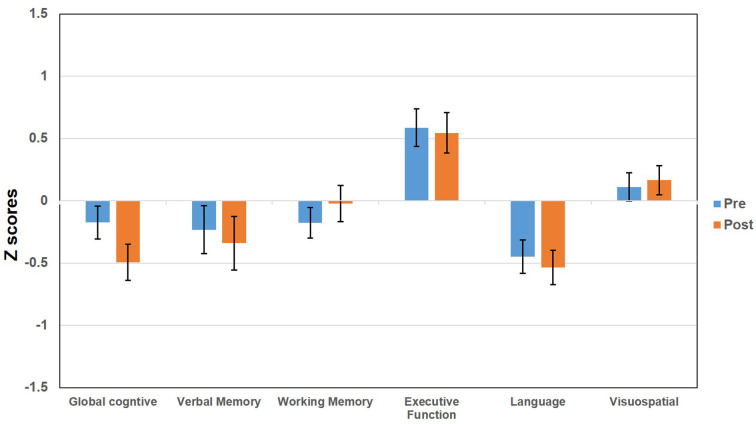
Bar graphs represent the pre and post deep brain stimulation (DBS) score (*z* scores) for essential tremor (ET) patients tested across multiple domains including global cognitive function, verbal memory, working memory, executive functioning, language, and visuospatial functioning. The error bars represent the standard error of the mean.

The baseline demographic and clinical characteristics of the ET patients included in the study are presented in Table 1. We included 50 ET patients (29 males, 21 females) who underwent DBS surgery at UF, had baseline neuropsychological assessments and presented for a follow-up 1 year after surgery. The mean ± SD for age at first DBS surgery was 68.18 (±10.1) years with a range of 34–83 years; the mean disease duration was 33.1 (±20.4) years with 10–60 years as the range; the mean duration of education was 14.2 (±3.5) years, and the mean tremor severity was 54.3 (± 14.7) before the surgery. There were 44 right-handed and six left-handed patients. There were 30 patients with left-brain implantation and seven patients with right-brain implantation and the goal was to control the arm tremor that was most bothersome to the patients. There were 13 patients with bilaterally staged surgeries used to target bilaterally significant symptoms. For these patients, the cognitive assessment performed 1 year after the second side implantation surgery was compared against the baseline assessment obtained before their first side was implanted. The anatomical coordinates for the active contacts relative to the mid-commissural points (MCP) were: 14.3 ± 1.6 (mm), lateral to midline; −4.3 ± 1.5 (mm) posterior to MCP; 2.1 ± 2.3 (mm) dorsal to intercommissural plane. The stimulation settings (mean ± SD and range) at 1-year follow-up after implantation were as follows: voltage (2.7 ± 0.7, 1.0–4.7), pulse width (102.3 ± 34.7, 60–210), and frequency (154.6 ± 23.7, 130–210). The values for two brain sides were averaged for individuals who had bilateral DBS surgeries. The TEED calculation was 123 μJ (±20.4) with the values for two brain sides averaged for bilateral DBS surgeries. There were no significant effects of age at surgery, duration of ET, baseline tremor severity, and TEED value calculations seen across all domains of cognitive outcomes. At baseline, on average the ET patients were intact on all cognitive domains with a relative weakness in language abilities (baseline mean = −0.43; SD = 0.9; Figure 1). We determined the cognitive functioning to be intact as the average performance of the cohort was generally closer to the mean normative values. Two patients could not complete the entire neuropsychology testing battery for the follow-up assessment because of time constraints and scheduling conflicts. Neuropsychologists performing the follow-up testing were blind to surgical complications, tremor ratings, and stimulation parameters.

**Table 1:** Clinical characteristics of essential tremor (ET) patients before deep brain stimulation (DBS) by DBS laterality.

	Left *n* = 30	Right *n* = 7	Bilateral *n* = 13		
	Mean (SD)	Mean (SD)	Mean (SD)	*F, χ^2^*	*P*-value
Age (years)	68.9 (9.0)	72.3 (8.4)	64.2 (12.5)	1.72	0.19
Education (years)	13.8 (3.6)	15.0 (2.9)	14.5 (3.6)	0.43	0.66
Duration of tremor (years)	32.7 (22.4)	41.3 (24.6)	30.2 (12.5)	0.62	0.54
Tremor Rating Scale total (years)	58.4 (15.5)	45.9 (9.7)	47.7 (9.9)	3.77	0.03
Dementia Rating Scale Total (*z* score)	−0.40 (0.89)	0.33 (0.94)	0.08 (0.84)	2.62	0.08
Working Memory Composite (*z* score)	−0.26 (0.68)	0.22 (0.80)	−0.22 (1.16)	0.93	0.40
Executive Composite (*z* score)	0.69 (1.14)	0.55 (0.84)	0.37 (0.95)	0.41	0.67
Verbal Memory Composite (*z* score)	−0.28 (1.24)	0.10 (1.47)	−0.30 (1.57)	0.24	0.79
Language Composite (*z* score)	−0.40 (0.99)	−0.77 (0.99)	−0.36 (0.70)	0.50	0.61
Visuospatial Composite (*z* score)	−0.04 (0.71)	0.08 (0.95)	0.45 (0.77)	1.88	0.17

### Cognitive Changes Following DBS

The analyses of pre-post changes among the entire sample revealed that there were no significant longitudinal changes for all cognitive domains included in the study (*p*-values range from 0.133 to 0.891).

### Cognitive Changes by Age at Tremor Onset

In our cohort, while the range for age of tremor onset was from five to 76 years of age, we found there was a bimodal distribution pattern with a median at 38 years. We, therefore, had two groups of patients; younger onset ET (<38 years) and older onset ET (>38 years).

For working memory, while the main effects were not significant, there was a significant interaction between time of assessment (pre vs. post) and age at onset (*F*_(1,47)_ = 4.47, *p* = 0.043). *Post hoc* testing revealed no significant change for the older onset ET, but the working memory improved for younger onset ET after DBS surgery. Except for working memory, there were no significant group differences in verbal memory, executive function, visuospatial function, language function, and general cognitive function.

### Cognitive Changes by DBS Type (Unilateral vs. Bilateral Lead Implantation)

There were 30 patients with left-brain implantation, seven patients with right-brain implantation, and 13 patients with bilateral surgeries. We combined the left and right brain to compare unilateral against bilateral DBS surgeries. We found, there were significant main effects of unilateral vs. bilateral DBS for general cognitive function (*F*_(1,50)_ = 2.71, *p* = 0.036) and visuospatial domain (*F*_(1,48)_ = 2.36, *p* = 0.01) but the main effects of time of assessment were not significant and there was no interaction between the time of assessment and assignment to unilateral vs. bilateral DBS. *Post hoc* pairwise comparisons revealed that the bilateral group scored higher in general cognitive function and visuospatial function than did the unilateral group.

There were no significant effects for the language, working memory, and executive function; however for verbal memory, while the main effects were not significant, there was a significant interaction effect between assessment time (pre vs. post) and unilateral vs. bilateral group (*F*_(1,45)_ = 5.20, *p* = 0.027). In the *post hoc* comparisons, bilateral DBS had higher verbal memory scores at post DBS evaluation compared to the unilateral; however, these comparisons only approached significance (*p* = 0.055).

### Cognitive Changes by Left vs. Right DBS (Laterality)

Within the subset of patients who underwent unilateral DBS, there were 30 left DBS patients and seven right DBS patients. Except for DRS, there were no significant effects observed for verbal and working memory, executive function, language, and visuospatial function. For the DRS, there was a significant main effect for the time of assessment (*F*_(1,37)_ = 4.61, *p* = 0.04) with a significant (*p* = 0.02) decline in score after surgery. However, there was no evidence for the effect of laterality or interaction.

### Cognitive Changes by Complications vs. No Complications

Five patients presented to our center with complications during and immediately after surgery comprising of stroke (*n* = 2, MCA territory hemisphere involved was nondominant and contralateral to the side of implantation), intracerebral hemorrhage (*n* = 1, small hemorrhage at the site of VIM target, resolved within 4 weeks), infection (*n* = 1, resolved with antibiotics), and subdural hemorrhage (*n* = 1, same side as implantation, resolved within 2 weeks). These patients had a complete clinical/radiological recovery within weeks of the onset of complications. When the cognitive outcomes were assessed, there was a significant interaction effect between assessment time and complications vs. non-complications group (*F*_(1,45)_ = 4.34, *p* = 0.043) for the verbal memory. In the *post hoc* comparisons that were Bonferroni-corrected, we found the verbal memory score decreased significantly (1.2 ± 0.9; *p* = 0.03) after surgery for those patients who developed complications after surgery. The remaining cognitive domains did not change significantly.

### Mood and Motor Changes After DBS Among ET Patients

Pre- and post-DBS changes in tremor severity (TRS), depression (BDI), and trait anxiety (STAI-T) were examined among ET patients. Results from repeated measure *T*-tests revealed a significant improvement in tremor severity following VIM DBS (the mean difference in TRS score = 26.4; *p* < 0.001). There were no significant changes in depression or trait anxiety after VIM DBS surgery even when subgroups analysis was performed (all *p* > 0.1).

## Discussion

Our ET DBS cohort that had intact neuropsychology assessment before the surgery and responded optimally to VIM stimulation at 6 months follow-up after surgery revealed no further cognitive decline at a 1-year follow-up. The neuropsychology assessment battery encompassed multiple cognitive domains, including global cognitive functions, executive functions, visuospatial functions, and language functions. Interestingly, working memory in patients who had tremor onset at a younger age improved after the surgery, but the verbal memory worsened in patients who developed complications during or immediately after the surgery albeit this was a small cohort.

Essential tremor is one of the most common movement disorders and is associated with cognitive impairments (even without DBS) that may be present in several domains (Bermejo-Pareja and Puertas-Martin, 2012). Tröster et al. (2002) found in a cohort of 101 ET patients, 25% scored below the standard cut-off for dementia in the Mattis Dementia Rating Scale. These patients revealed lower than average scores on measures of complex auditory and visual attention, response inhibition, executive functions, verbal fluency, and immediate recall of a word list. In another study, Duane and Vermilion (2002) found executive functioning and visual attention was impaired in 28% of patients. Deficits in attention and visuoperception were also confirmed in two further studies when extended psychometric batteries were applied (Higginson et al., 2008; Kim et al., 2009). These cognitive symptoms likely represent the disruption of the cerebellar-thalamocortical pathways projecting to the frontal lobe (Bermejo-Pareja and Puertas-Martin, 2012).

In patients with ET who receive DBS therapy, it would not be surprising to see the worsening of cognition given that trajectory of the lead when targeted to the VIM nucleus in the thalamus traverses the frontal lobe and in some patients may traverse the caudate nucleus (Heber et al., 2013), and thalamus has a definite role in cognitive functioning. However, there is limited data on the cognitive impact of VIM DBS in ET with many studies that do not support this theory (Table 2). In one of the earlier studies, Tröster et al. (1999) found statistically significant but clinically modest gains on tasks of visuoperceptual and constructional ability, visual attention, delayed word list recognition, and prose recall at 3 months follow-up after surgery. There was a slight worsening of lexical verbal fluency. The same group presented their findings of a longer follow-up of 12 months that showed improvements in on a cognitive screening measure and in aspects of verbal memory, fine visuomotor, and visuoperceptual functions (Fields et al., 2003). Heber et al. (2013) found the cognitive domains of memory, executive and intellectual functions and verbal fluency did not worsen even 6 years after DBS surgery. In another study of 17 ET DBS patients, verbal fluency, both semantic and phonemic was worse when VIM was stimulated at high-frequency (120–150 Hz) compared to low-frequency (10 Hz). However, there were no differences observed between high-frequency stimulation and when DBS was turned off (Pedrosa et al., 2014).

The overall nature of stable cognitive outcomes observed in our study and many other studies may be a selection bias since there was an inclusion of patients who did not have significant deficits before DBS surgery. The effects may therefore be possibly dependent on the baseline cognitive reserves. Fields et al. (2003) found the verbal fluency to decline significantly in 4/40 patients however these patients had diminished verbal fluency before they received the DBS, suggesting that a baseline deficit may have predisposed them to experience a further decline. The decline in ET DBS verbal fluency in the Fields study was similar to that seen in patients with Parkinson’s disease. Finally, the word output was found to decrease after bilateral DBS implantation surgeries (Ehlen et al., 2016) whereas, in another study that mainly comprised of unilateral DBS surgeries, language processing was impaired at the level of syntax however the speed, rates of errors, word classes, and lexical diversity were largely unaffected (Ehlen et al., 2017).

**Table 2:** Summary of ET DBS studies.

Reference	*N*	Target	Surgical details	Length of Follow-up	Outcomes and comments
Tröster et al. (1999)	40	VIM	Unilateral	3 months	Stable cognition
Woods et al. (2003)	49	VIM	Unilateral	3 months	Tremor onset >37 years and high pulse width settings associated with worsening cognition
Fields et al. (2003)	40	VIM	Unilateral	1 year	Stable cognition; verbal fluency declined in 10% patients, these patients had baseline deficits
Heber et al. (2013)	9	VIM	Unilateral	6 years	Stable cognition
Pedrosa et al. (2014)	17	VIM	Bilateral	4.6 years	Stable cognition; High frequency compared to low frequency stimulation worsened verbal fluency but no difference seen when compared to off stimulation
Ehlen et al. (2016)	13	VIM	12 unilateral, 1 bilateral	3.5 years	language change; more use of para tactical compared to syntactic structure
Ehlen et al. (2017)	13	VIM	Bilateral	2.8 years	Decrease in word output
Klein et al. (2017)	26	VIM	Bilateral	2 years	Stable cognition
Fytagoridis et al. (2013)	17	CZI	Unilateral	1 year	slight decline in semantic verbal fluency
Philipson et al. (2019)	26	CZI	20 Unilateral, 6 bilateral	2 year	decline in verbal fluency

*VIM, ventral intermedius nucleus; CZI, caudal zona incerta*.

Recently, DBS studies in ET have focused on stimulation of caudal zona incerta (ventral to the VIM region) stimulation beyond the VIM as the two structures are common to the tremor circuitry involved in ET. Fytagoridis et al. (2013) found while DBS adversely influenced the verbal fluency immediately after surgery when compared against a baseline, this effect dissipated at 1 year both on and off stimulation suggesting the deterioration likely reflected a micro-lesion effect. In a study involving caudal zona incerta stimulation, although the overall cognitive functioning was stable, there was a slight but statistically significant decline in semantic fluency at a 1-year follow-up (Philipson et al., 2019).

A few studies have also shown improvement of cognitive functioning for ET with DBS besides the earlier work by Tröster et al. (1999). In one study, there was an improvement of simple reaction time when the DBS was turned on (Heber et al., 2013). In another study, there were improvements in attention and general cognitive functions (Klein et al., 2017). Our study found while the age at surgery had no significant influence, the working memory improved after DBS in ET patients who developed tremors at a younger age. The working memory performance was likely not influenced by other factors such as mood and anxiety levels that remained stable through the length of follow-up.

Although we did not find a group level change in verbal memory with DBS, we found persistently impaired verbal memory scores on the Hopkins Verbal learning test and Wechsler Memory Scale III scale in patients who developed surgical complications. The complication rate seen in our group was similar to that published by other large DBS studies (Rezai et al., 2006; Voges et al., 2006), and in our study, persistent cognitive impairment was observed despite a complete clinical recovery. Verbal memory was also observed to remain unaffected after DBS by other investigators at long-term follow-up (Heber et al., 2013). Fields et al. (2003) reported improvements in verbal memory assessed with the California Verbal Learning scale and the Wechsler Memory revised scale in addition to those seen in general cognitive functions, visuomotor, and visuoperceptual functions at 1-year follow-up. However, these studies did not examine the influence of surgical complications. It has long been known that the thalamus plays an important role in explicit memory (Thomas and Gash, 1985). Specifically, the ventral anterior (VA) nuclei of the thalamus have been involved in the encoding of information, and the medial dorsal (MD) nuclei of the thalamus play a role in the retrieval of information (Van der Werf et al., 2003). Future studies should examine the contribution of these nuclei to memory performance following VIM DBS.

We acknowledge our study did not include a DBS naïve ET control group; most patients in the cohort had unilateral DBS implantation, and the length of follow-up was limited. Additionally, the current study was unable to address the exact influence of medication dosages and the extent that cognitive changes could be a persistent lesion effect or a stimulation effect (i.e., examining memory performance among post-DBS patients who do not have the stimulator “turned on”). However, our study does report the cognitive outcomes from one of the largest cohorts to date and includes a comprehensive neuropsychological test battery encompassing all aspects of cognitive assessment. Our study included several relevant DBS related factors including the electrical energy delivered with stimulation.

In summary, the cognitive outcomes with VIM DBS in our cohort were stable likely related to the selection of patients with well-preserved cognitive reserves at baseline. There were isolated gains in working memory for younger onset ET and circumscribed decline in verbal memory for patients who developed surgical complications, but the underlying basis for these effects will be better understood if the future studies include imaging-based tractography to shed insights into the specific fiber pathways that are affected by stimulation.

## Data Availability Statement

The original contributions presented in the study are included in the article, further inquiries can be directed to the corresponding author.

## Ethics Statement

The studies involving human participants were reviewed and approved by IRB University of Florida. The patients/participants provided their written informed consent to participate in this study.

## Author Contributions

JJ, TO, WH, ZJ, DB, SC, AR-Z, KF, MO, and AW fulfilled the authorship criteria by substantial contributions to the conception of the work, providing data for the work, revisiting it critically for important intellectual content, approving the final version, and agreeing to be accountable for all aspects of the work in ensuring that questions related to the accuracy or integrity of any part of the work are appropriately investigated and resolved.

## Conflict of Interest

The authors declare that the research was conducted in the absence of any commercial or financial relationships that could be construed as a potential conflict of interest.
